# Modelling of XCO_2_ Surfaces Based on Flight Tests of TanSat Instruments

**DOI:** 10.3390/s16111818

**Published:** 2016-11-01

**Authors:** Li Li Zhang, Tian Xiang Yue, John P. Wilson, Ding Yi Wang, Na Zhao, Yu Liu, Dong Dong Liu, Zheng Ping Du, Yi Fu Wang, Chao Lin, Yu Quan Zheng, Jian Hong Guo

**Affiliations:** 1 State Key Laboratory of Resources and Environment Information System, Institute of Geographical Sciences and Natural Resources Research, Chinese Academy of Sciences, Beijing 100101, China; zhangll@lreis.ac.cn (L.L.Z.); jpwilson@dornsife.usc.edu (J.P.W.); zhaon@lreis.ac.cn (N.Z.); liuyu.15b@igsnrr.ac.cn (Y.L.); duzp@lreis.ac.cn (Z.P.D.); wangyf@lreis.ac.cn (Y.F.W.); gjh@irsa.ac.cn (J.H.G.); 2 Spatial Sciences Institute, Dana and David Dornsife College of Letters, Arts, and Sciences, University of Southern California, Los Angeles, CA 90089-0374, USA; 3 Ministry of Education Key Laboratory for Nonequilibrium Synthesis and Modulation of Condensed Matter, School of Science, Xi’an Jiaotong University, Xi’an 710049, China; dingyiwang@hotmail.com (D.Y.W.); liudongdongzhijia@163.com (D.D.L.); 4 Changchun Institute of Optics, Fine Mechanics and Physics, Chinese Academy of Sciences, Changchun 130033, China; linchaoluck@163.com (C.L.); zhengyq@sklao.ac.cn (Y.Q.Z.)

**Keywords:** TanSat, flight test, XCO_2_ retrieval, HASM, XCO_2_ simulation

## Abstract

The TanSat carbon satellite is to be launched at the end of 2016. In order to verify the performance of its instruments, a flight test of TanSat instruments was conducted in Jilin Province in September, 2015. The flight test area covered a total area of about 11,000 km^2^ and the underlying surface cover included several lakes, forest land, grassland, wetland, farmland, a thermal power plant and numerous cities and villages. We modeled the column-average dry-air mole fraction of atmospheric carbon dioxide (XCO_2_) surface based on flight test data which measured the near- and short-wave infrared (NIR) reflected solar radiation in the absorption bands at around 760 and 1610 nm. However, it is difficult to directly analyze the spatial distribution of XCO_2_ in the flight area using the limited flight test data and the approximate surface of XCO_2_, which was obtained by regression modeling, which is not very accurate either. We therefore used the high accuracy surface modeling (HASM) platform to fill the gaps where there is no information on XCO_2_ in the flight test area, which takes the approximate surface of XCO_2_ as its driving field and the XCO_2_ observations retrieved from the flight test as its optimum control constraints. High accuracy surfaces of XCO_2_ were constructed with HASM based on the flight’s observations. The results showed that the mean XCO_2_ in the flight test area is about 400 ppm and that XCO_2_ over urban areas is much higher than in other places. Compared with OCO-2’s XCO_2_, the mean difference is 0.7 ppm and the standard deviation is 0.95 ppm. Therefore, the modelling of the XCO_2_ surface based on the flight test of the TanSat instruments fell within an expected and acceptable range.

## 1. Introduction

Atmospheric carbon dioxide (CO_2_) is the dominant anthropogenic greenhouse gas, and a crucial factor in global climate change [1]. However, our current knowledge about the temporal and spatial variability of atmospheric CO_2_ is still insufficient, which leads to large uncertainties in our estimates of carbon sources and sinks [2,3,4]. Ground-based observations, for example, are accurate but too spatially sparse to sufficiently infer CO_2_ sources and sinks [3,5,6,7,8,9]. Space-based measurements, on the other hand, have the potential to yield global estimates of the distribution of the atmospheric CO_2_ vertical column (in molecules/cm^2^) or the CO_2_ dry air column-averaged mole fraction (in ppm), especially in areas not covered by ground-based observations [5,10].

Near and short-wave infrared (NIR) measurements have sufficiently high precision and sensitivity to the planetary boundary layer, where the largest signals of sources and sinks occur [2,3,6,7,11,12,13,14,15,16]. The first NIR carbon satellite instrument, SCIAMACHY, onboard ENVISAT that was launched in 2002 and lost in 2012, measured reflected solar radiation in the NIR spectral bands [4,9]. Presently, there are only two NIR carbon satellite instruments orbiting the Earth, which enable the retrieval of XCO_2_ with significant sensitivity in the boundary layer (i.e., near the Earth’s surface). The two instruments are TANSO, onboard GOSAT launched in 2009 and OCO-2 launched in 2014 [12,13,14]. China’s first satellite to monitor atmospheric XCO_2_, TanSat, will be launched at the end of 2016. A flight test was conducted in September, 2015 to evaluate the performance and stability of TanSat’s instruments for acquiring high resolution absorption spectra, and to reduce the risk of TanSat operating problems after launch. Simultaneous ground-based measurements of geophysical parameters were also collected and can be used to evaluate the accuracy of the retrieved atmospheric XCO_2_ estimates_._

The remainder of this article is structured as follows: Section 2 introduces the flight area and data used in this article. Section 3 introduces the methods used in this study, including the deployment of a full physics retrieval algorithm to retrieve XCO_2_ in the flight test area, the use of regression modeling to estimate the approximate XCO_2_ surface, and the use of the HASM platform to model the XCO_2_ surface. Section 4 analyzes the retrieval results and distribution of XCO_2_ in the flight test area. Section 5 concludes the paper and discusses future work.

## 2. Study Area and Data

### 2.1. Flight Logistics and Data

TanSat aims to deliver XCO_2_ in ppm at a high spatial resolution in square grids measuring 3 km on a side. An evaluation of the performance of the TanSat imaging spectrometer prototype was conducted as part of flight tests on the 11th, 14th and 16th of September, 2015. The flight demand parameters, such as the flight height, flight speed, flight time, solar zenith angle, flight geometric requirements and meteorological conditions, can be seen in Table 1.

The flight’s spectrometer covered two of the three NIR spectral bands that will be included in the final TanSat spectrometer (Table 2). We can see that band 1 observed the O_2_ A-band spectral region (758–773 nm) at 0.044 nm spectral resolution and that Band 2 covers the 1592–1625 nm spectral region with a spectral resolution of 0.13 nm. The third TanSat spectral band (2060 nm), which will provide CO_2_ absorption and water vapour information, was not included in the prototype and flight test due to cost.

**Figure 1:**
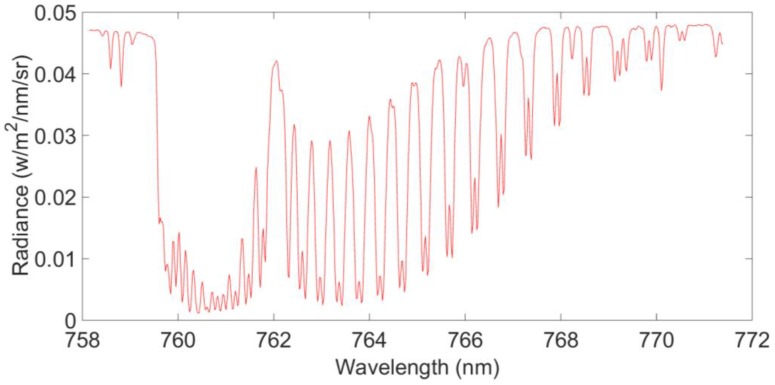
Band 1’s flight test observational L1B data: spectral radiance in 760 nm.

**Figure 2:**
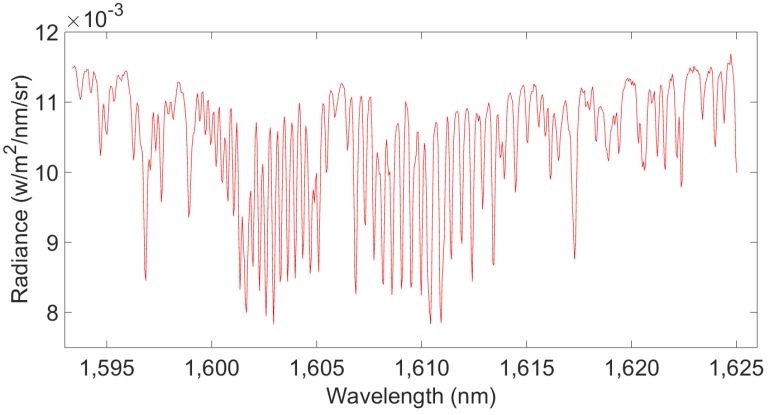
Band 2’s flight test observational L1B data: spectral radiance in 1610 nm.

For this study, we acquired the flight test’s L1B data (calibrated and geographically located spectral radiances) from the Changchun Institute of Optics, Fine Mechanics and Physics, Chinese Academy of Sciences. They had processed the dark background and calibrated the spectral radiation for the test flight’s L1A DN (digital number) data. Samples of two NIR spectral bands derived from the L1B data used in the study can be seen in Figure 1 and Figure 2. The X-axis shows spectral wavelengths (in nm), and the Y-axis represents the spectral radiance (in W/m^2^/nm/sr). Band 1 contains O_2_ absorption information and can be used to estimate the O_2_ column with high near-surface sensitivity. Similarly, band 2 contains CO_2_ weak absorption information and can be used to estimate the CO_2_ column with high near-surface sensitivity.

### 2.2. Study Area and Surface Characteristics

**Figure 3:**
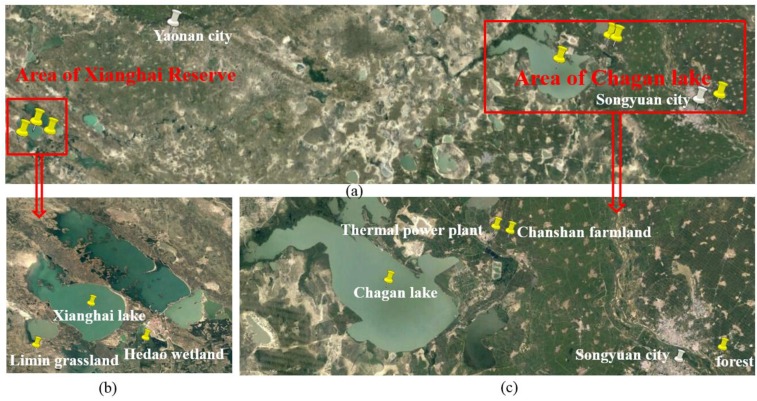
The flight test area showing: (**a**) the overall flight test area; (**b**) Xianghai Reserve; and (**c**) the Changan Lake area.

The flight test passed over Xianghai Reserve and Chagan Lake in Jilin Province (Figure 3). The underlying surface included several lakes, forest, grass, and wetlands, farmland, a thermal power plant and numerous cities and villages. The test area runs from 122.205° E to 124.946° E and from 44.980° N to 45.360° N and cover an area of about 11,000 km^2^. The left red box of Figure 3 covers the Xianghai Reserve and includes the Limin grassland, Hedao wetland and Xianghai Lake. The right red box of Figure 3 shows the Chagan Lake area and includes the Chanshan town thermal power plant, farmland, Chagan Lake and Songyuan City.

Ground synchronous observations were also collected during the flight period, and provided important auxiliary data for XCO_2_ retrieval and simulation in the flight test area. The observations and methods used to gather them are listed in Table 3. Temperature, humidity, pressure and wind profiles were obtained by sonde measurements and used to summarize the state of the atmosphere for XCO_2_ retrieval and surface estimation (Table 3). The CO_2_ concentration at the surface was observed with a Greenhouse gas online laser analyzer. The CO_2_ profile gathered with a captive balloon and CO_2_ concentration in the surface layer were used as dependent variables in the flight test area when we established the approximate XCO_2_ surface. Aerosol optical depth and surface reflectance are also crucial factors for XCO_2_ retrieval and were observed with a sun photometer and ASD spectrometer, respectively.

## 3. Methods

**Figure 4:**
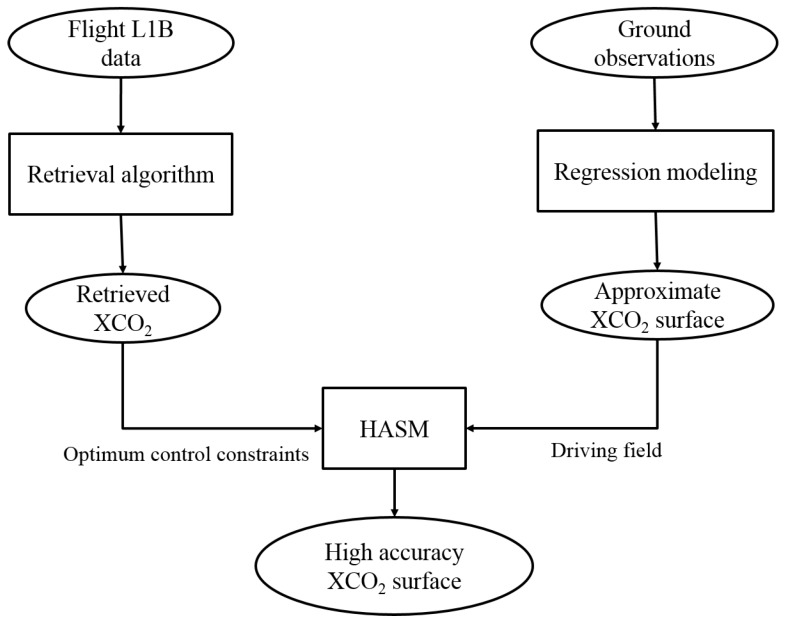
The overall workflow.

We first retrieved the XCO_2_ using a full physics retrieval algorithm. However, it is difficult to directly analyze the spatial distribution of XCO_2_ in the flight test area using the limited retrieval results. The high accuracy surface modeling (HASM) method has been widely used in the simulation of climate change, CO_2_ concentrations in the surface layer, and terrestrial land cover change [17,18,19]. We therefore used HASM to fill the gaps where there was no information on XCO_2_ in the flight test area. The entire workflow reproduced in Figure 4 includes three main steps. First, we used a full physics retrieval algorithm to retrieve XCO_2_. Second, we used regression modeling to establish the approximate (i.e., not very accurate) surface of XCO_2_ in the flight test area. And third, we used HASM, which took the approximate surface of XCO_2_ as its driving field and retrieved XCO_2_ as its optimum control constraints, to simulate a high accuracy XCO_2_ surface for the whole flight test area.

### 3.1. Retrieval Algorithm

**Figure 5:**
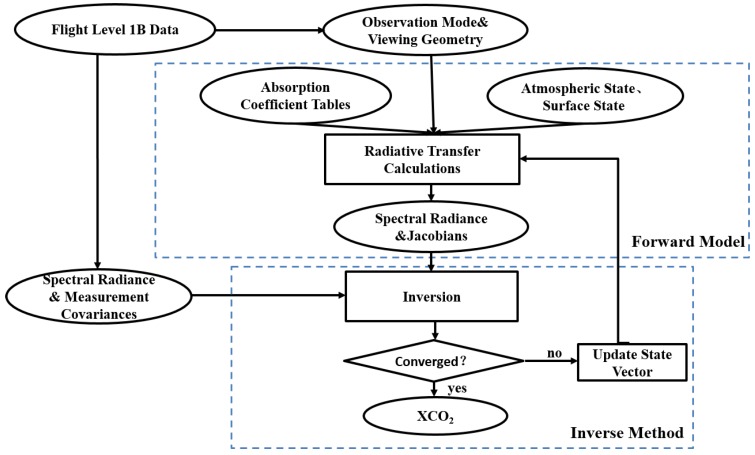
Major components and tasks included in the retrieval algorithm workflow.

In this study, the retrieval algorithm is primarily used for simultaneously fitting the O_2_ A-band at 760 nm and the CO_2_ band at 1610 nm to get column abundances of CO_2_ and O_2_ based on the optimal estimation method [20]. The retrieval algorithm is an iterative retrieval method which calls the radiative transfer model with updated parameters after each iteration step. The primary components of the flight XCO_2_ retrieval processing workflow are summarized in Figure 5. Given that the atmospheric and surface states and the observing geometry for a sounding, the forward model generates radiance spectra and Jacobians (as will be described in more detail in Section 3.1.1 below). This model first generates two synthetic spectra that fully resolves the solar spectrum, the absorption and scattering cross sections for each atmospheric gas and the reflecting surface. The inverse method is based on Rodger’s optimal estimation approach. This method modifies the initial state vector to minimize differences between the observed and simulated spectra from each sounding. This inverse method is described in more detail in Section 3.1.2 below. 

#### 3.1.1. Forward Model

Synthetic spectra were generated using a radiative transfer algorithm. Losses can be caused by absorption and the scattering of the atmosphere. The sources (i.e., gains) mainly come from atmospheric emission and multiple scattering, and the general expression of the radiative transfer is given by the following equation [21]:

(1) $$\mu \frac{dI(\tau ,\mu )}{d\tau }=I(\tau ,\mu )-J(\tau ,\mu )$$

where μ is the cosine of the zenith angle, *I* is the specific intensity, *J* is the source function (multiple scattering), and τ is the optical depth.

The radiative transfer model SCIATRAN was used to solve Equation (1). SCIATRAN is a comprehensive software package for the modeling of radiative transfer processes in the terrestrial atmosphere and ocean in the spectral range from the ultraviolet to the thermal infrared (i.e., 0.18–40 μm) including multiple scattering processes, polarization, thermal emission and ocean-atmosphere coupling. The software is capable of modeling spectral and angular distributions of the intensity or the Stokes vector of the transmitted, scattered, reflected, and emitted radiation assuming either a plane-parallel or a spherical atmosphere [22].

The inputs and outputs of the SCIATRAN model can be seen in the Table 4. The solar irradiance spectra were acquired from Dr. Kurucz (http://kurucz.harvard.edu). Gas absorption and scattering cross sections were obtained from the SCIATRAN software package which incorporates a climatological database obtained from a 2D chemical transport model. The atmospheric state (temperature, humidity and pressure profiles), surface state and aerosol optical properties were taken from ground synchronous observations and the European Centre for Medium-Range Weather Forecasts (ECMWF) reanalysis data. A set of instrument line shape functions were provided by the Changchun Institute of Optics, Fine Mechanics and Physics, Chinese Academy of Sciences. The outputs of the forward model were synthetic spectra and Jacobians.

#### 3.1.2. Inverse Method

The inversion was used to obtain the unknown geophysical parameters (CO_2_ and O_2_ profiles) which minimize the differences between the observed and synthetic spectra from each sounding. The inverse method is based on Rodger’s optimization algorithm in which the spectrum is expressed symbolically as in Equation (2):

(2) y=F(x,b)+∈

where *x* is the unknown state vector, *b* is a set of quantities required by the forward model but not retrieved, *F* is the forward model, and ∈ are the spectral errors due to the measurements and the forward model.

To find the state vector with the maximum a posteriori probability, we minimized the cost function (Equation (3)):

(3) $$\chi^{2}={\left(y-F\left(x,b\right)\right)}^{T}S_{\in }^{-1}\left(y-F\left(x,b\right)\right)+{\left(x-x_{a}\right)}^{T}S_{a}^{-1}\left(x-x_{a}\right)$$

where $S_{\in }$ is the error covariance matrix corresponding to the measurement vector, $x_{a}$ is the a priori state vector which holds the prior knowledge about the state vector elements and $S_{a}$ is the corresponding a priori error covariance matrix which specifies the uncertainties of the a priori state vector elements as well as their cross-correlations.

The Levenberg-Marquardt method was used to obtain the solution in an iterative manner (Equation (4)):

(4) $$x_{i+1}=x_{i}+\tilde{S}[K_{i}^{T}S_{\in }^{-1}\left(y-F\left(x_{i}-b\right)\right)-S_{a}^{-1}\left(x_{i}-x_{a}\right)]$$

(5) $$\tilde{S}={\left(K_{i}^{T}S_{\in }^{-1}K_{i}+\left(1+\gamma \right)S_{a}^{-1}\right)}^{-1}$$

where *K* is the Jacobian or weighting function matrix consisting of the derivatives of the forward model with respect to the state vector elements *K* = ∂*F*(*x*,*b*)/∂*x*. In the case of convergence, $x_{i+1}$ is the most probable solution given the measurements and prior knowledge and it is then denoted as the maximum a posteriori solution $\tilde{x}$ of the inverse problem. $\tilde{S}$ is the corresponding covariance matrix consisting of the variances of the retrieval state vector elements and their correlations. The damping factor γ adjusts the step size of the iteration in a way which ensures that each step further minimizes the cost function.

The inverse method continued to provide iterative improvements of the solutions until both of convergence criteria listed below were achieved and we could obtain CO_2_ and O_2_ column abundances:

(1) The fitted residuals with root mean square error (RMSE) differences between synthetic and observation spectra less than some pre-determined threshold: 0.25% for CO_2_ and 2% for O_2_ window [4,13].
(2) The normalized successive difference of the state vector is less than some pre-determined threshold (1%) in the TANSO-FTS SWIR L2 algorithm (see [2] for details).

For CO_2_ we derived column-averaged dry air mole fractions by normalizing the CO_2_ columns with the simultaneously retrieved oxygen columns retrieved from the O_2_ A-band. Oxygen is an accurate proxy for the air column because its mole fraction is well known and has negligibly small variations. Then XCO_2_ was calculated using Equation (6) [4,23] as follows:

(6) $$\mathrm{XC}O_{2}=\frac{CO_{2}^{col}}{o_{2}^{col}/o_{2}^{mf}}$$

where $CO_{2}^{col}$ is the retrieved absolute CO_2_ column (in molecules/cm^2^), $o_{2}^{col}$ is the retrieved absolute O_2_ column (in molecules/cm^2^), and $o_{2}^{mf}\;\left(0.2095\right)$ is the assumed (column-averaged) mole fraction of O_2_ used to convert the O_2_ column into a corresponding dry air column.

### 3.2. Derivation of Approximate XCO_2_ Surface 

**Figure 6:**
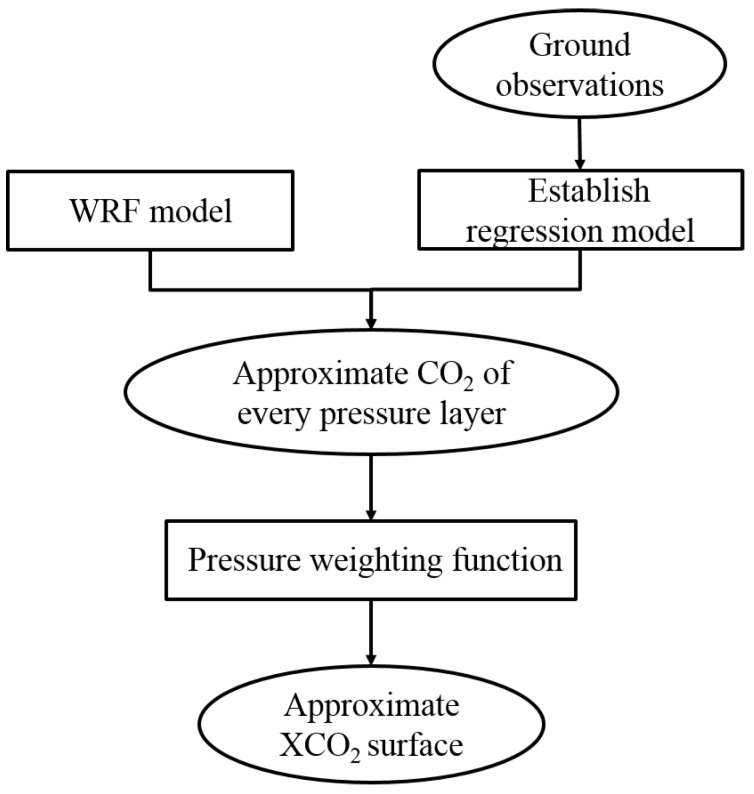
Workflow used to estimate approximate XCO_2_ surface.

The approximate XCO_2_ surface in the flight test area was required to establish as the driving field of HASM (described in Section 3.3 below). The processing steps used to estimate the approximate XCO_2_ are summarized in Figure 6. Firstly, we used the least squares fit for the captive balloon’s CO_2_ profiles, the surface CO_2_ concentration in the surface layer and other synchronous ground observations to build a regression model [17,18]. Secondly, we combined the regression model with the Weather Research and Forecasting (WRF) model [24,25] to derive the approximate CO_2_ surface for every pressure layer in the whole flight test area. Finally, the pressure weighting function was used to merge every pressure layer’s CO_2_ surface to estimate the approximate XCO_2_ surface.

#### 3.2.1. Initial Regression Model

We used an ordinary least squares multivariate linear fit method to build the regression models. The two groups of explanatory variables listed in Table 5 were used to build separate regression models for the CO_2_ concentrations in the surface layer [18] and the CO_2_ profile. We used the modeling spatial relationships/ordinary least square tools in ArcGIS to perform this work, and both models passed the F test at the 95% confidence level.

The CO_2_ concentration in every pressure layer at every grid point in the flight test area was then calculated as follows:

(7) $$Y_{CO2}=b+\overset{n}{\underset{i=1}{\sum }}a_{i}x_{i}$$

where $Y_{CO2}$ is the CO_2_ concentration in every pressure layer, b is a constant, *x* represents the explanatory variables, *a* is the corresponding coefficient for each of the explanatory variables, and *n* is the number of explanatory variables.

#### 3.2.2. WRF Model

WRF is a next-generation mesoscale numerical weather prediction system designed for both atmospheric research and operational forecasting needs. The model serves a wide range of meteorological applications across scales from tens of meters to thousands of kilometers. WRF can generate atmospheric simulations using real-world data or idealized conditions. WRF offers operational forecasting a flexible and computationally-efficient platform for operational forecasting using initial data from the US National Center for Environmental Prediction (NCEP). 

The initial data used in this study were derived from reanalysis datasets of NCEP. We generated forecasts at 20:00 each day using an integrate interval of 30 h and 1 km by 1 km grid. Physical parameters were the YSU scheme, Monin Obukhov scheme, WSM 6-class graupel scheme, RRTM scheme (longwave), Goddard scheme (shortwave), Noah-MP land-surface model and Kain-Fritsch (new Eta) scheme respectively. We used WRF Version 3.5 to model the CO_2_ concentration at every point using the aforementioned explanatory variables in the flight test area on an hourly basis from 10:00 to 14:00 on the 11th, 14th, and 16th of September. We then used Equation (7) to calculate the approximate CO_2_ concentration of every pressure layer at each point in the flight test area.

#### 3.2.3. Pressure Weighting Function

The pressure weighting function h relates the local CO_2_ concentration specified on the discrete pressure levels to the profile-weighted average [8], so XCO_2_ could be calculated as follows:

(8) $${\mathrm{XCO}}_{2}=\overset{n}{\underset{i=1}{\sum }}h_{i}u_{i}$$

where *u* denotes the CO_2_ concentration and the subscripts refer to the layers, and:

(9) $$h_{i}=\left|\left(-p_{i}+\frac{p_{i+1}-p_{i}}{\ln(p_{i+1}/p_{i})}\right)+\left(p_{i}-\frac{p_{i}-p_{i-1}}{\ln(p_{i}/p_{i-1})}\right)\right|\frac{1}{p_{surf}}$$

where *p* denotes the pressure and the subscripts again refer to the layers, and $p_{surf}$ denotes the surface pressure. For the edge layers, if *i* = 1, only the left first term applies, that is $h_{1}=\left|\left(-p_{1}+\frac{p_{2}-p_{1}}{\ln(p_{2}/p_{1})}\right)\right|\frac{1}{p_{surf}}$, while if *i* = *n*, only the left second term applies, that is $h_{n}=\left|\left(p_{n}+\frac{p_{n}-p_{n-1}}{\ln(p_{n}/p_{n-1})}\right)\right|\frac{1}{p_{surf}}$.

The approximate CO_2_ concentration for every pressure layer at each point in the flight test area was calculated using the methods described in Section 3.2.1 and Section 3.2.2. With the methods specified at this step, the approximate XCO_2_ was obtained by averaging the approximate CO_2_ concentration of every pressure layer, weighted by the pressure weighting function.

### 3.3. High Accuracy Surface Modeling 

The high accuracy surface modeling [26] platform takes global approximate information (e.g., remote sensing images or model simulation results) as its driving field and local accurate information (e.g., ground observation and/or sampling data) as its optimum control constraints. A surface can be uniquely defined by the first and the second fundamental coefficients [27,28,29,30,31] in terms of the fundamental theorem of surfaces. The first fundamental coefficients are used to express the intrinsic geometric properties that do not depend on the shape of the surface, but only on measurements that we can carry out while on the surface itself. The second fundamental coefficients reflect the local warping of the surface, namely its deviation from a tangent plane at the point under consideration, which can be observed from outside the surface. The Earth’s surface system or a component surface of the Earth’s surface environment can be simulated with HASM when its spatial resolution is fine enough and is uniquely defined by both extrinsic and intrinsic invariants of the surface.

If a surface is a graph of a function z=f(x,y), the first fundamental coefficients *E*, *F* and *G* can be formulated as

(10) $$\{\begin{array}{c} E=1+f_{x}^{2}\\ G=1+f_{y}^{2}\\ F=f_{x}\cdot f_{y} \end{array}$$

The second fundamental coefficients *L*, *M* and *N* can be formulated as

(11) $$\{\begin{array}{c} L=\frac{f_{xx}}{\sqrt{1+f_{x}^{2}+f_{y}^{2}}}\\ N=\frac{f_{yy}}{\sqrt{1+f_{x}^{2}+f_{y}^{2}}}\\ M=\frac{f_{xy}}{\sqrt{1+f_{x}^{2}+f_{y}^{2}}} \end{array}$$

The first and the second fundamental coefficients should satisfy the following Gauss equation set:

(12) $$\left\{ \begin{array}{l} f_{xx}=\Gamma_{11}^{1}\cdot f_{x}+\Gamma_{11}^{2}\cdot f_{y}+L\cdot {\left(E\cdot G-F^{2}\right)}^{-1/2}\\ f_{yy}=\Gamma_{22}^{1}\cdot f_{x}+\Gamma_{22}^{2}\cdot f_{y}+N\cdot {\left(E\cdot G-F^{2}\right)}^{-1/2}\\ f_{xy}=\Gamma_{12}^{1}\cdot f_{x}+\Gamma_{12}^{2}\cdot f_{y}+M\cdot {\left(E\cdot G-F^{2}\right)}^{-1/2} \end{array}\right.$$

where $\Gamma_{11}^{1}=\frac{1}{2}\left(G\cdot E_{x}-2F\cdot F_{x}+F\cdot E_{y}\right)\cdot {\left(E\cdot G-F^{2}\right)}^{-1}$, $\Gamma_{12}^{1}=\frac{1}{2}\left(G\cdot E_{y}-F\cdot G_{x}\right)\cdot {\left(E\cdot G-F^{2}\right)}^{-1},$ $\Gamma_{22}^{1}=\frac{1}{2}\left(2G\cdot F_{y}-G\cdot G_{x}-F\cdot G_{y}\right)\cdot {\left(E\cdot G-F^{2}\right)}^{-1}$, $\Gamma_{11}^{2}=\frac{1}{2}\left(2E\cdot F_{x}-E\cdot E_{y}-F\cdot E_{x}\right)\cdot {\left(E\cdot G-F^{2}\right)}^{-1}$, $\Gamma_{12}^{2}=\frac{1}{2}\left(E\cdot G_{x}-F\cdot E_{y}\right)\cdot {\left(E\cdot G-F^{2}\right)}^{-1}$, $\Gamma_{22}^{2}=\frac{1}{2}\left(E\cdot G_{y}-2F\cdot F_{y}+F\cdot G_{x}\right)\cdot {\left(E\cdot G-F^{2}\right)}^{-1}$ are the Christoffel symbols of the second kind which depend only on the first fundamental coefficients and their derivatives.

Finite difference methods are used for solving the Gauss equation set (Equation (12)). It can be simplified as the following equation set [26]:

(13) $$\{\begin{array}{c} A\cdot z^{\left(n+1\right)}=d^{\left(n\right)}\\ B\cdot z^{\left(n+1\right)}=q^{\left(n\right)}\\ C\cdot z^{\left(n+1\right)}=p^{\left(n\right)} \end{array}$$

If $f_{i,j}$ is the value of z=f(x,y) at the pth sampled point $\left(x_{i},y_{i}\right)$ in the computational domain, the simulation value should be equal or approximate to the sampling value of this lattice so that the constraint equation is added to the simplified equation set (Equation (13)). The matrix formulation of the HASM master equations can be expressed as follows [26]:

(14) $$\left[A^{T}\;B^{T}\;C^{T}\;\lambda \cdot S^{T}\right]\left[\begin{array}{c} A\\ \begin{array}{c} B\\ C\\ \lambda \cdot S \end{array} \end{array}\right]z^{\left(n+1\right)}=\left[A^{T}\;B^{T}\;C^{T}\;\lambda \cdot S^{T}\right]\left[\begin{array}{c} d^{\left(n\right)}\\ \begin{array}{c} q^{\left(n\right)}\\ p^{\left(n\right)}\\ \lambda \cdot k \end{array} \end{array}\right]$$

where the parameter λ is the weight of the sampling points and determines the contribution of the sampling points to the simulated surface. λ could be a real number, which means all sampling points have the same weight, or a vector, which means every sampling point has its own weight. An area affected by a sampling point in a complex region is smaller than in a flat region. Therefore, a smaller value of λ is selected in a complex region and a bigger value of λ is selected in a flat region.

High accuracy surface modeling can simulate continuous attribute variations in three-dimensional space, and has been successfully used in simulating climate change [17,32,33], constructing DEMs [28,29], interpolating soil properties [34,35,36] and terrestrial land cover change [19]. For this study, HASM took the approximate XCO_2_ surface as its driving field and the flight retrieved XCO_2_ as its optimum control constraints as shown in Equation (15):

(15) Xsim=HASM(Xinitial,Xsam)

where Xsim is the final XCO_2_ surface which is calculated by HASM, Xinitial is the approximate XCO_2_ surface which is obtained by regression modeling, and Xsam is the retrieval XCO_2_. The main inputs of HASM are the retrieval XCO_2_ and the approximate XCO_2_ surface with the output being the high accuracy surface of XCO_2_.

## 4. Results 

### 4.1. Retrieval XCO_2_

**Figure 7:**
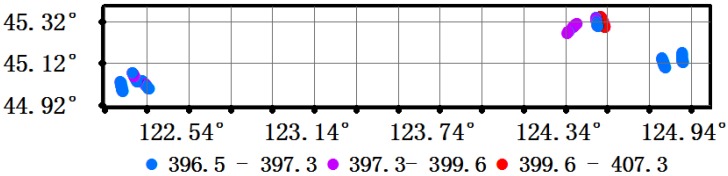
XCO_2_ concentrations estimated in the flight test area using full physics retrieval approach.

The XCO_2_ concentrations estimated with the full physics retrieval approach for about 400 points in the flight test area covering seven kinds of underlying surface—grassland, water, wetland, farmland, a thermal power plant, urban area and forest land—are shown in Figure 7.

The retrieval XCO_2_ concentrations ranged from 396.5 to 407.3 ppm with a mode of 396.5 to 397.3 ppm (Figure 7).

### 4.2. Approximate XCO_2_ Surface in the Flight Test Area

**Figure 8:**
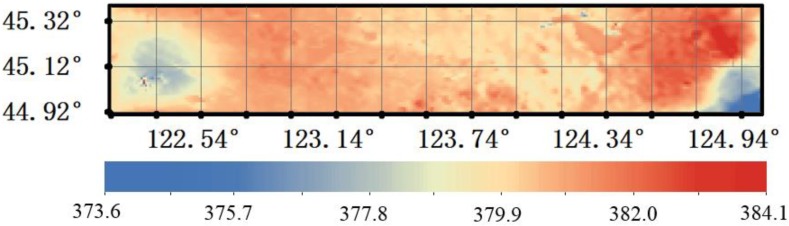
Approximate XCO_2_ surface in the flight test area.

In order to model the high accuracy XCO_2_ surface, we first established the approximate XCO_2_ surface using an ordinary linear least squares regression model to represent the spatial relationships in the flight test area (Figure 8).

From Figure 3 and Figure 8, we can see that the mean XCO_2_ of this approximate surface is about 20 ppm lower than the retrieval XCO_2_ surface, but does show the estimated spatial relationships in the flight test area. It was used as the driving field of HASM, and the retrieval XCO_2_ was used as the optimum control constraints to model the XCO_2_ surface in the flight test area. 

### 4.3. High Accuracy XCO_2_ Surface 

**Figure 9:**
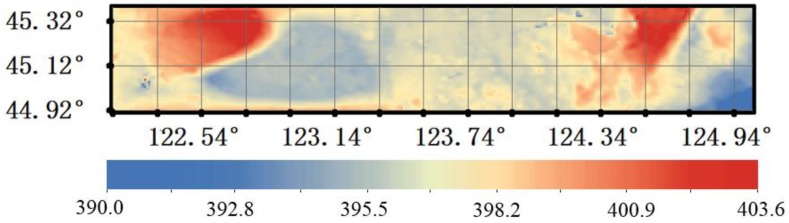
The XCO_2_ concentrations in the flight test area estimated with HASM.

The high accuracy surface model took the regression results as its driving field and the retrieval XCO_2_ values as its optimum control constraints to more accurately estimate the XCO_2_ surface in the whole flight test area (Figure 9). 

The HASM XCO_2_ estimates in the flight test area ranged from 390.0 to 403.6 ppm. Combining the map in Figure 9 with the image showing the flight test area (Figure 3), we can see that XCO_2_ is highest in Songyuan City. Similarly, the XCO_2_ in another small city, Taonan, is higher as well. The XCO_2_ was lower in the Xianghai Reserve, which includes the Limin grassland, Hedao wetland and Xianghai Lake. The aforementioned results show how human activities can increase XCO_2_ and that the retrieval XCO_2_ calculated in this study was sensitive to the boundary layer which may be affected by human’s activities over relatively short distances.

### 4.4. Comparison with OCO-2’s XCO_2_ Estimates

**Figure 10:**
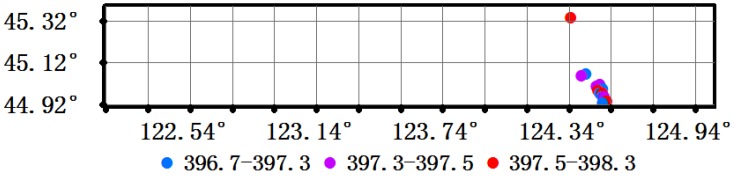
The XCO_2_ estimates from OCO-2 in the flight test area.

The Orbiting Carbon Observatory 2 (OCO-2) which was launched on 2 July 2014, is NASA’s first dedicated Earth remote sensing satellite to study XCO_2_ from Space. OCO-2 is collecting space-based global measurements of XCO_2_ with the precision, resolution, and coverage needed to characterize sources and sinks on regional scales. OCO-2 is also able to quantify CO_2_ variability over seasonal cycles year after year. The OCO-2 instrument flies on a dedicated spacecraft and includes three high-resolution grating spectrometers which make coincident measurements of reflected sunlight in the near-infrared CO_2_ near 1610 and 2060 nm and in the molecular oxygen (O_2_) A-Band at 760 nm at a resolution of 1.29 (longitude) and 2.25 km (latitude). The XCO_2_ (Version OCO2_L2_Lite_FP.7r) at the 14 locations in the flight test area ranged from 396.7 to 398.3 ppm on 11–16 September 2014 (Figure 10).

The corresponding HASM XCO_2_ estimates were extracted from Figure 9 and used to calculate the differences from the OCO-2 XCO_2_ estimates (Table 6).

Twelve of the 14 HASM XCO_2_ estimates were higher than the corresponding OCO-2 estimates (column 6, Table 6) with the mean difference and standard deviation being 0.7 and 0.95 ppm, respectively. Given that the accuracy of the OCO-2 XCO_2_ estimates is about 1 ppm [37], the HASM modeling results based on the flight test of the TanSat instruments fell within the expected range.

## 5. Conclusions

This paper presents the validation of XCO_2_ based on a flight test of TanSat instruments using a combination of full physics and surface modelling. The results produced with this pair of methods were nevertheless sufficiently similar to justify using HASM to estimate the XCO_2_ surface. HASM took the approximate surface of XCO_2_ as its driving field and the flight’s retrieval XCO_2_ as its optimum control constraints to fill in the gaps across the whole flight test area. The results showed that the XCO_2_ in cities is higher than in other places suggesting that cities are places where human activities can lead to increased XCO_2_ concentrations. Compared with OCO-2’s XCO_2_, the mean difference is 0.7 ppm and the standard deviation is 0.95 ppm. Due to the good matching, the surface modelling of XCO_2_ based on the flight test of TanSat instruments fell within an expected and accepted range.

The flight test in this study was conducted in good weather conditions, so we assumed the sky was clear when the retrieval XCO_2_ estimates were acquired. In fact, the presence of clouds and aerosols could influence XCO_2_ and in the future, we plan to consider their impact. Moreover, the CO_2_ strong absorption band (i.e., 2060 nm) contains additional information on CO_2_ and H_2_O, but we did not use this band for the current flight test due to cost. Therefore, we will conduct XCO_2_ retrieval using three bands from other satellite data before TanSat is launched in the future.

## Figures and Tables

**Table 1:** Flight parameters.

Type	Parameters
Flight height	5 km ± 30 m
Flight speed	220 ± 6.6 km/h
Flight time	10:30–13:30
Solar zenith angle	40°–55°
Flight geometric requirements	Flight drift angle is <5°; range of three-axis attitude angle is <2°; route is straight and course deviation is <60 m.
Meteorological conditions	Good weather with visibility >10 km.

**Table 2:** NIR spectral bands used for the flight retrieval.

	Band 1	Band 2
Wavelength range (nm)	758–772	1592–1625
Spectral resolution (nm)	0.044	0.13

**Table 3:** List of ground observations and methods used to gather them.

Observations	Observation Methods Used
Temperature profile	Sonde measurement
Humidity profile	Sonde measurement
Pressure profile	Sonde measurement
Wind profile	Sonde measurement
CO_2_ profile	Captive balloon
CO_2_ concentration in surface layer	Greenhouse gases online laser analyzer UGGA
Surface reflectance	Analytical Spectral Devices (ASD) spectrometer
Aerosol optical depth	Sun photometer CE318

**Table 4:** SCIATRAN model inputs and outputs.

Inputs	Outputs
Solar irradiance spectra	Radiance spectrum
Gas absorption and scattering cross sections	Jacobians (partial derivatives of the radiance spectrum with respect to each of the state vector elements)
Atmospheric state	-
Surface state	-
Instrument line shape function	-
Aerosol optical properties	-

**Table 5:** Explanatory variables used to model CO_2_ in different layers.

Dependent Variables	Explanatory Variables
CO_2_ concentration in surface layer	Surface pressure, atmospheric humidity, atmospheric temperature, soil humidity, soil temperature, upward and downward shortwave radiation, altitude, longitude and latitude
CO_2_ profile (not including surface layer)	Temperature, pressure and humidity profiles, wind speed and direction, latitude and longitude

**Table 6:** The XCO_2_ difference between HASM and OCO-2 estimates in the flight test area.

Number	Longitude (°)	Latitude (°)	Flight Test (ppm)	OCO-2 (ppm)	Difference (ppm)
1	124.35	45.34	396.67	398.34	−1.67
2	124.40	45.06	399.13	397.54	1.59
3	124.42	45.07	398.85	397.07	1.78
4	124.47	45.01	398.05	397.35	0.70
5	124.48	45.00	398.36	397.49	0.87
6	124.48	44.99	398.05	397.84	0.21
7	124.49	45.02	398.75	397.42	1.33
8	124.49	44.98	397.82	397.25	0.57
9	124.50	45.00	399.01	397.23	1.78
10	124.50	44.98	398.73	397.63	1.10
11	124.50	44.96	397.99	396.73	1.26
12	124.50	44.93	397.24	397.04	0.20
13	124.51	44.96	397.92	397.29	0.63
14	124.52	44.94	397.35	397.87	−0.52
